# An Invertebrate Burn Wound Model That Recapitulates the Hallmarks of Burn Trauma and Infection Seen in Mammalian Models

**DOI:** 10.3389/fmicb.2020.00998

**Published:** 2020-06-03

**Authors:** Evgenia Maslova, Yejiao Shi, Folke Sjöberg, Helena S. Azevedo, David W. Wareham, Ronan R. McCarthy

**Affiliations:** ^1^Division of Biosciences, Centre for Inflammation Research and Translational Medicine, Department of Life Sciences, College of Health and Life Sciences, Brunel University London, London, United Kingdom; ^2^School of Engineering and Materials Science, Institute of Bioengineering, Queen Mary, University of London, London, United Kingdom; ^3^The Burn Centre, Department of Hand and Plastic Surgery, Linköping University, Linköping, Sweden; ^4^Department of Clinical and Experimental Medicine, Faculty of Health Sciences, Linköping University, Linköping, Sweden; ^5^Antimicrobial Research Group, Blizard Institute, Queen Mary, University of London, London, United Kingdom

**Keywords:** burn, infection, *Galleria mellonella*, *Pseudomonas aeruginosa*, MRSA, *Acinetobacter baumannii*, biofilm

## Abstract

The primary reason for skin graft failure and the mortality of burn wound patients, particularly those in burn intensive care centers, is bacterial infection. Several animal models exist to study burn wound pathogens. The most commonly used model is the mouse, which can be used to study virulence determinants and pathogenicity of a wide range of clinically relevant burn wound pathogens. However, animal models of burn wound pathogenicity are governed by strict ethical guidelines and hindered by high levels of animal suffering and the high level of training that is required to achieve consistent reproducible results. In this study, we describe for the first time an invertebrate model of burn trauma and concomitant wound infection. We demonstrate that this model recapitulates many of the hallmarks of burn trauma and wound infection seen in mammalian models and in human patients. We outline how this model can be used to discriminate between high and low pathogenicity strains of two of the most common burn wound colonizers *Pseudomonas aeruginosa* and *Staphylococcus aureus*, and multi-drug resistant *Acinetobacter baumannii.* This model is less ethically challenging than traditional vertebrate burn wound models and has the capacity to enable experiments such as high throughput screening of both anti-infective compounds and genetic mutant libraries.

## Introduction

Burn wound infection is one of the main clinical complications associated with burn wound care and is a leading cause of mortality among burn wound patients (Branski et al., 2009; Calum et al., 2017; Akers et al., 2019). Burn wound infections are also associated with autograft failure and prolonged treatment regimes, placing a significant burden on global health care systems. Specific nosocomial pathogens have established the burn unit as a particular niche in which they thrive; this is seen with clonal outbreaks occurring in burn units around the world. The primary burn wound pathogens include *Pseudomonas aeruginosa*, *Acinetobacter baumannii*, and *Staphylococcus aureus* (Branski et al., 2009; Azzopardi et al., 2011; Fransen et al., 2016; Amissah et al., 2017). Due to the complexity of the damage induced by thermal injuries, the insights offered by *in vitro* or *ex vivo* models are limited (Abdullahi et al., 2014). This has led to the development of different animal models to study burn wound infections. These models primarily use mammals such as mice, rats, dogs, and pigs. Murine models have become well established as the principal model to study burn wound infections (Everett et al., 2017; Lenzmeier et al., 2019). These murine models can offer robust insights into the host/pathogen processes involved in burn wound colonization and can play a key role in the validation of novel therapeutic strategies. However, they are governed by strict ethical guidelines, associated with high levels of suffering and are limited by the numbers of animals that can be used in a given study. Another complication with mouse burn models is that mice are classified as “loose-skin animals,” meaning that they do not have a similar skin structure to humans and the process of wound healing is different. The process of burn healing in mice is contraction, whereas in humans, it is granulation and re-epithelization (Wong et al., 2011; Abdullahi et al., 2014). The porcine model has emerged as a viable alternative to the mouse model particularly when studying wound healing as there is considerable similarity of skin structure, immune response, and healing pattern (re-epithelization) between pigs and humans (Abdullahi et al., 2014; Roy et al., 2016; Tsai D. M. et al., 2016). However, studies are limited to eight to nine experimental animals, due to high maintenance costs and strict ethical procedures, which limits statistical power and infection output kinetics. Although mammalian models have indisputable advantages, they are ethically challenging and not amenable to large cohort studies or drug discovery strategies.

*Galleria mellonella*, larvae of the greater wax moth, have become an increasingly popular non-mammalian infection model (Andrea et al., 2019). This is largely due to the exclusion of invertebrates from the Animals (Scientific Procedures) Act 1986, simplifying the ethical approval for research. *G. mellonella* are now well established as a powerful model to study pathogenicity in a range of bacterial and fungal pathogens (Fedhila et al., 2010; Tsai C. J. et al., 2016; McCarthy et al., 2017a,b). In comparison to other mammalian models, *G. mellonella* models of infection have a number of distinct advantages: the larvae are easy to use without special equipment, are inexpensive, and can be maintained at a range of temperatures including 37°C. Larvae are also readily available from a range of sources including facilities where they are reared under controlled conditions (genotype, age, weight, diet) specifically for scientific use. *G. mellonella* also have a similar innate immunity to mammalian models with primitive immune cells called hemocytes, which are capable of phagocytosis and producing a range of comparable innate immune effectors (Lange et al., 2018). In this study, we outline for the first time a *G. mellonella* burn wound model that can be used to study burn trauma and wound infection.

## Materials and Methods

### Bacterial Strains

*Pseudomonas aeruginosa* (PA14, PA14Δ*esxA*), *Staphylococcus epidermidis* ATCC 12228 (American Tissue Culture Collection), methicillin resistant *S. aureus* (MRSA, National Collection of Type Cultures 12493), and *A. baumannii* (AB5075) were used. Single colonies were inoculated in lysogeny broth (LB) and incubated at 37°C for 16–18 h at 180 r/min before the experiment.

### *G. mellonella* and Burn Wound Creation

**FIGURE 1:**
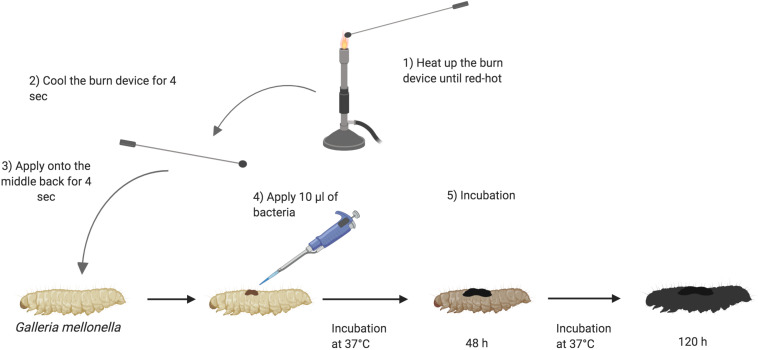
Schematic outline of burn procedure: (1) Heating up the burn device in the middle of the flame of a Bunsen burner until the metal element is red/white-hot. (2) Cooling down the burn device near the flame for 4 s. (3) Applying the hot metal element to the middle segments of *Galleria mellonella* back for 4 s and slowly lifting it. (4) Applying 10 μl of 1:10 dilution of over-night bacterial culture onto the fresh wound. (5) Larvae are incubated at 37°C for 120 h and monitored every 24 h. The darkness of *Galleria* signifies the intensity of infection (Wojda, 2017). Created with BioRender.

*Galleria mellonella* were sourced from Biosystems Technology (Exeter, United Kingdom) or LiveFood UK Ltd. (Somerset, United Kingdom). The animals were at a stage in their life cycle where they do not need to be fed. They are stored at 4°C. Prior to use, larvae were sorted into Petri dishes lined with Whatman filter paper (Fisher, United Kingdom), 10 larvae per plate and stored at 4°C until use. The larval bodies were sterilized with 70% ethanol. The burn was induced with a heated steel instrument to achieve a burn consistent burn area of approximately 2 mm^2^. The metal instruments used were of low thermal conductivity and specifically selected to generate reproducible burn wounds. The instrument was heated until it was red/white-hot and applied to the middle section of the back of larvae for a fixed time of 4 s (Figure 1). This location was chosen so the wound could be easily visualized without having to physically manipulate the larvae. For total burn surface area experiments, the number of times the burn instrument was applied to the larval back depended on the size of burn wound desired. For infection studies, immediately post burn, the wound was inoculated with 10 μl of 1:10 dilution of bacterial over-night culture. For assays using *A. baumannii*, a single colony was applied to the wound. Any larvae who showed distress or leakage of hemolymph after the burn process were immediately euthanized by incubating at -20°C for 20 min to minimize suffering. Post wounding, larvae were incubated at 37°C and monitored over the course of 72–120 h (3–5 days). Mortality was recorded by complete melanization of the larval body and complete loss of motility (McCarthy et al., 2017b).

### Fluid Resuscitation

Larvae were burned three times as previously described. 15 min post burn wound, larvae were injected in the right-hand side of the first set of prolegs with 100 μl of sterile phosphate buffered saline (PBS) solution using Micro Fine Insulin syringe 0.5 ml 29 G × 12.7 mm. Larvae were incubated at 37°C and monitored over the course of 72 h (3 days). Mortality was recorded by complete melanization of the larval body and complete loss of motility.

### Bacteremia Assays

To determine bacterial dissemination from the burn wound site into the circulation (hemolymph), immediately after death, 10 μl of hemolymph was aspirated from larvae. This hemolymph was serially diluted in PBS solution to a dilution of 10^–6^ and then 20 μl from each dilution was plated on *Pseudomonas* Isolation Agar (Thermo Fisher^®^). Plates were incubated at 37°C overnight and colony forming units enumerated.

### Statistical Analysis

Data was generated from a minimum of 10 larvae per condition per biological replicate with between two to four biological replicates per experiment. Kaplan–Meier survival curves were used to visualize data and a Log Rank test performed with *p* < 0.05 considered significant. For CFU counting assays, a Mann–Whitney *U*-test was performed with *p* < 0.05 considered significant.

## Results

### *G. mellonella* Burn Surface Area Is Linked to Survival Prognosis

**FIGURE 2:**
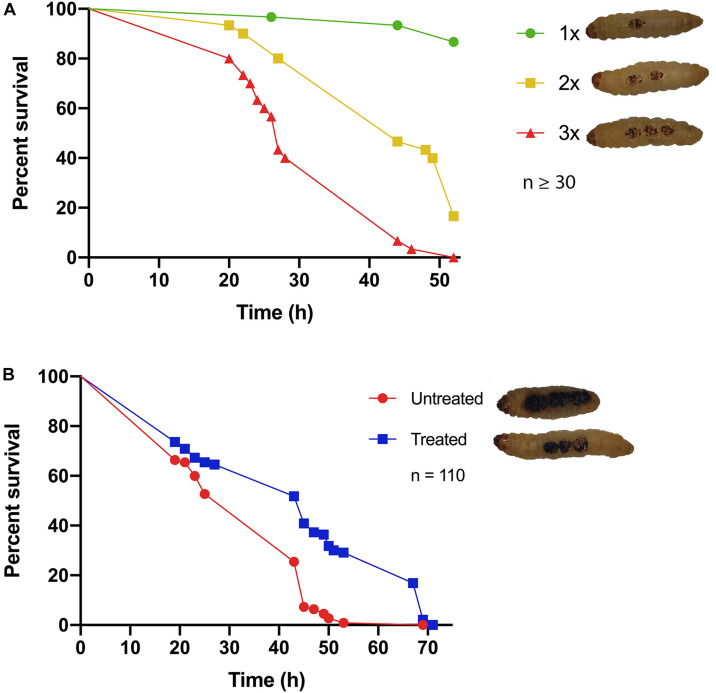
Hallmarks of burn trauma: **(A)** Total burn surface area (TBSA) correlates with survival: it is well established in burn patients that the larger the TBSA, the greater the risk of mortality. We demonstrate that this central dogma can be recapitulated in the *G. mellonella* burn wound model. 1x = 2 mm^2^ Burn, 2x = 2 × 2 mm^2^ Burn, 3x = 3 × 2 mm^2^ Burns. 1x vs 2x, Log Rank *p* < 0.001, 1x vs 3x Log Rank *p* < 0.001, 2x vs 3x Log Rank *p* < 0.001. *n* ≥ 30 per condition. **(B)** Fluid resuscitation: administering larvae with fluid after burn trauma can significantly increase their survival prognosis Log Rank *p* < 0.001. *n* ≥ 30 per condition.

Classical burn wound studies in mammalian models typically use a heated metal instrument to achieve consistency in burn area [TBSA% (percentage total body surface area)] and burn intensity (superficial versus deep) (Tsai D. M. et al., 2016). To determine if *G. mellonella* were a suitable model to study burn trauma and burn wound infection, a protocol was developed to mimic mammalian models and create a localized area of thermal tissue injury, without causing the excessive loss of hemolymph, the larval equivalent of blood, from the burn wound site (Figure 1). The burn injury procedure is well tolerated by larvae, as in the control group (1x Burn only), the percentage of live larvae was >87% after 52 h at 37°C (Figure 2A), highlighting this invertebrate as a potential model of burn trauma. In human burn treatment, it is recognized that the increase in total burn surface is directly linked to a decrease in survival probability (Kraft et al., 2012). To determine if this paradigm is also true in the *G. mellonella* model, we tested the impact of increased burn wound surface area on survival. Sequentially increasing the total burn surface area was shown to lead to a corresponding decrease in survival with less than 20% of larvae with double the burn surface area surviving for 52 h (Log Rank, *p* < 0.001), whereas 0% of larvae with triple the burn surface survived to this time point (Log Rank, *p* < 0.001) (Figure 2A).

### Survival Can Be Increased Through Fluid Resuscitation

It is widely recognized that treating severe fluid loss is the greatest problem faced by clinicians when dealing with major burn injuries. Therefore, fluid resuscitation is one of the central cornerstones of modern burn treatment regimens (Baxter and Shires, 1968; Baxter, 1979; Haberal et al., 2010). Early observations in our assay suggested that the larvae that did succumb to burn trauma, did so due to severe fluid loss. This suggested that the fluid loss paradigm may hold true in the *G. mellonella* model. To test this, 15 min after receiving a fatal burn wound, larvae were injected with 100 μl PBS and their survival was monitored. There was a significant difference in survival between larvae that received fluid resuscitation and untreated larvae (Log Rank, *p* < 0.001), confirming that severe fluid loss is at least partly responsible for mortality in this model (Figure 2B). This event mimics the fluid loss in human burn injury.

### *P. aeruginosa* Can Colonize *G. mellonella* Burn Wounds Leading to Mortality

**FIGURE 3:**
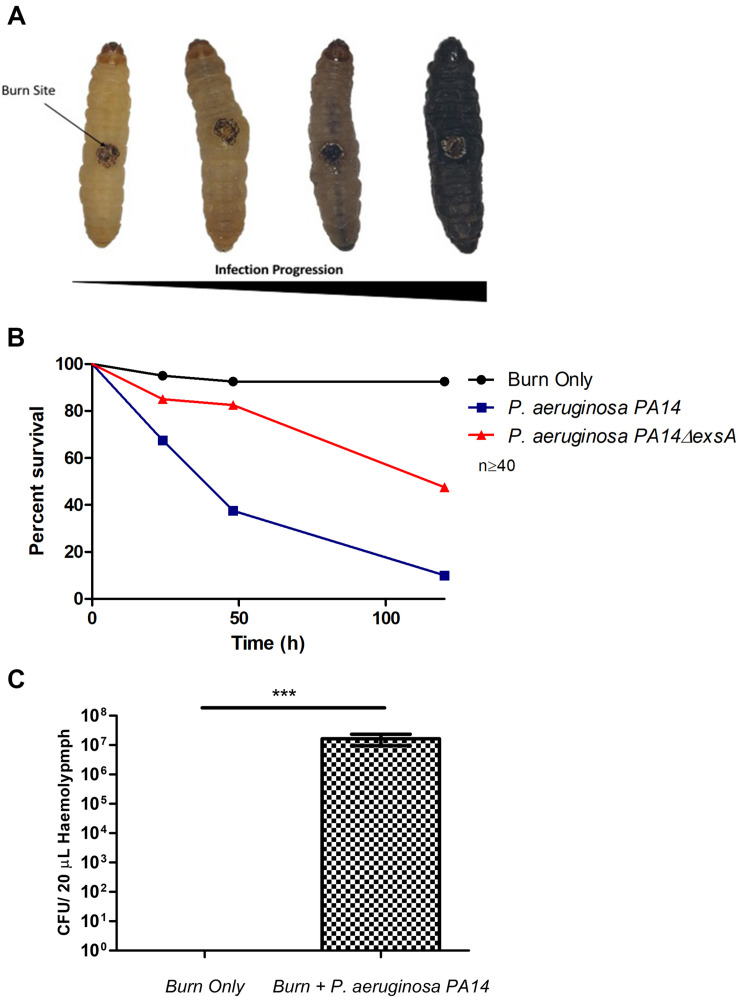
Visualization of burn wound pathogenicity over time: **(A)** Following the burn wound procedure and subsequent infection, the tissue at site of the burn will turn necrotic and evidence of bacterial dissemination is seen through the melanization of the larvae and loss of motility. Representative images of each stage shown. **(B)** Percentage survival of larvae after infecting the burn site with PA14Δ*exsA* which has an inactive T3SS and Wild Type PA14 against the uninfected control group burn. PA14 vs Burn Only Log Rank *p* < 0.001, Burn Only vs PA14Δ*exsA* Log Rank *p* < 0.001, PA14Δ*exsA* vs Burn Only Log Rank *p* < 0.001. *n* ≥ 40 per condition. **(C)** Hemolymph (larval blood) was aspirated and the bacterial numbers enumerated by plating serial dilutions *n* = 25 larvae, with SEM. Mann–Whitney *U*-test ***p < 0.001.

*Pseudomonas aeruginosa* is the most common Gram-negative burn wound pathogen and despite the remarkable advances in human burn patient care over the last century, patients who become infected with *P. aeruginosa* face a mortality rate of up to 80% (Tredget et al., 2004). To determine if *P. aeruginosa* could colonize larval burn wounds, larvae were topically infected with *P. aeruginosa* PA14, a known highly pathogenic burn wound isolate (Mikkelsen et al., 2011; McCarthy et al., 2014). This inoculation method mimics the exposure route that has been shown to occur in both the environment and burn clinics (Kolmos et al., 1993; Calum et al., 2017). Larvae infected with *P. aeruginosa* PA14 displayed high levels of mortality with only 10% survival observed after 120 h (Log Rank, *p* < 0.001) (Figures 3A,B and Supplementary Figure S1). The observed infection progression cycle in this model closely matches what is seen in mammalian models and human cases, with local tissue necrosis, biofilm formation, dissemination from the burn wound site, bacteremia, burn sepsis, and ultimately mortality.

To verify that this observed mortality was due to bacterial pathogenicity, a mutant strain of PA14 was used in which the master regulator of the Type 3 Secretion System (T3SS), *exsA* was deleted. Previous studies have demonstrated that inactivating the T3SS significantly diminishes the capacity of *P. aeruginosa* to infect murine burn wounds (Holder et al., 2001). Exposure of PA14Δ*exsA* to the larval burn wound resulted in significantly lower levels of mortality compared to the Wild Type PA14 (Log Rank, *p* < 0.001) (Figure 3B and Supplementary Figure S1), replicating the scenario seen in the mouse burn wound model (Holder et al., 2001).

**FIGURE 4:**
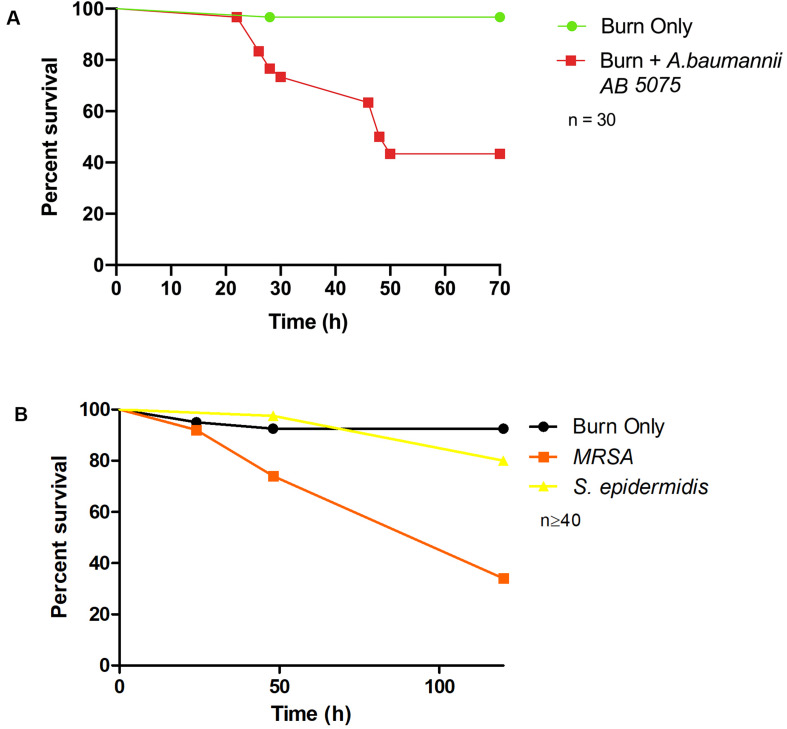
Burn wound pathogenicity: **(A)** Percentage survival rate of larvae infected with *A. baumannii* AB5075 and the burn only control. *A. baumannii* AB5075 vs Burn Only Log Rank *p* < 0.001. **(B)** Percentage survival rate of larvae infected with MRSA and the non-burn wound pathogen *S. epidermidis*. MRSA vs Burn Only Log Rank *p* < 0.001, MRSA vs *S. epidermidis* Log Rank *p* < 0.001, *S. epidermidis* vs Burn Only Log Rank *p* < 0.14. *n* ≥ 30 per condition.

To demonstrate bacterial dissemination from the burn wound site and bacteremia, hemolymph, the larval equivalent of blood, was aspirated from larvae immediately post-mortem. *P. aeruginosa* was recovered from the hemolymph of these larvae in 92% of cases whereas in the burn only control, *P. aeruginosa* was never recovered (Figure 3C). This further demonstrates that the infection progression cycle mimics what is observed in mammalian burn wound models and human patients. To determine if this model could be used to study other Gram-negative burn wound pathogens such as *A. baumannii*, the assay was repeated using a highly virulent multidrug resistant strain of *A. baumannii* AB5075 (Jacobs et al., 2014). As expected, larvae infected with *A. baumannii* AB5075 demonstrated a significant drop in survival compared to burn only controls (Log Rank, *p* < 0.001) (Figure 4A).

### MRSA Can Cause Mortality in the *G. mellonella* Burn Wound Model

To further explore the application of this model to the study of burn wound infections, we tested the capacity of a common Gram-positive pathogen MRSA to cause mortality in this model (Nishiguchi et al., 2017). MRSA is known to be a common burn wound pathogen and is frequently identified in burn care centers (Amissah et al., 2017). Although not as virulent as *P. aeruginosa*, MRSA could cause a significant decrease in burn wound survival compared to the uninfected controls with only 34% survival after 120 h (Log Rank, *p* < 0.001) (Figure 4B). To further interrogate the capacity of *Staphylococcus* spp. to cause mortality in this model and demonstrate the role of biofilm formation in infection progression, a strain of *S. epidermidis* (ATCC 12228) that does not readily form biofilm and has not previously been reported to cause burn wound infections was tested. Exposure of this strain to the burn site did not result in a significant increase in mortality compared to the uninfected burn wound control (Figure 4B).

## Discussion

Burn wounds are complex microenvironments where colonization by bacterial pathogens such as *P. aeruginosa*, *A. baumannii*, and *S. aureus* represents the primary clinical complication. Infection is the leading cause of mortality among burn wound patients (Branski et al., 2009; Calum et al., 2017). Burn wound infections are also associated with increased autograft failure and prolonged treatment regimes. This places a significant burden on burn treatment centers. The problem of burn wound infection is exacerbated by the emergence of multidrug resistance (MDR) among bacterial isolates, commonly found in the hospital setting, with one study reporting that 73% of septic deaths in a pediatric burn center were due to MDR organisms (Williams et al., 2009). Current *in vivo* models to study burn trauma and infection are exclusively based on mammalian models such as mice, rats, and pigs. The ability to gain *in vivo* insights into burn wound infection or test novel therapeutic options is hampered by the difficulty in gaining ethical approval for these mammalian models. This is due to the severity of the procedures involved and the limited quantities of data that can be realistically retrieved from these experiments. We describe for the first time an invertebrate model of burn trauma and infection. We demonstrate that this model closely mimics the hallmarks of burn trauma seen in patients such as a decreased survival prognosis with increased burn surface area and the importance of rapid fluid resuscitation (Figures 2A,B), hallmarks which cannot be reproduced in *in vitro* burn wound models (Figures 2A,B).

Substantial improvements in our understanding of burn trauma and infection and the development of effective treatment regimens have had a dramatic impact on patient survival. Despite this, the mortality rate for patients who become infected with *P. aeruginosa* has been unchanged in the last 25 years (Tredget et al., 2004; Calum et al., 2017). *P. aeruginosa* burn wound infections are notoriously difficult to treat and while new insights are consistently emerging into the molecular mechanisms that control *P. aeruginosa* burn wound colonization (Gonzalez et al., 2016; Everett et al., 2017), the lack of unrestricted *in vivo* models has stymied the progress in understanding this pathogen. We show that *P. aeruginosa* PA14 can colonize *G. mellonella* burn wounds and that this infection progression cycle is remarkably similar to infection progression in patients and mammalian models (Figures 3A,B). The transition from burn wound colonization to blood stream colonization is a critical event, which significantly reduces the probability of survival (Drake and Montie, 1988; Everett et al., 2017). We demonstrate that this model can also be used to study this event, as *P. aeruginosa* can be recovered from larval hemolymph to indicate that the bacteria have disseminated from the wound site (Figure 3C). One of the primary virulence mechanisms utilized by *P. aeruginosa* to establish infection is the T3SS. This system is capable of injecting a range of potent toxins, ExoS, ExoT, ExoY, and ExoU, into eukaryotic cells. Strains of *P. aeruginosa* with a defective T3SS are severely attenuated for virulence in a wide range of virulence models including pneumonia, neutropenic, corneal, and burn wound infection models (Finck-Barbançon et al., 1997; Sawa et al., 1998; Holder et al., 2001; Koh et al., 2005; Lee et al., 2005; Dortet et al., 2018; Williams McMackin et al., 2019). Critically, all of these infection models are mammalian models, we demonstrate for the first time that burn wound pathogenesis in the *G. mellonella* infection model is also severely attenuated in a strain with a defective T3SS (Figure 3B).

We demonstrate that this model can also be used to study *A. baumannii*, one of the leading proponents of the emergent antibiotic resistance crisis and a leading cause of burn wound infections. This pathogen can thrive in the hospital environment due to its remarkable ability to survive desiccation. Uniquely, *A. baumannii* can survive on inanimate objects such as hospital beds and ventilators without nutrients or water for weeks to months, which promotes transmission throughout health care environments (Farrow et al., 2018; Harding et al., 2018; Lima et al., 2018). We have demonstrated the versatility of this model by showing that it is suitable to also study Gram-positive burn wound pathogens such as MRSA.

Biofilm formation plays a central role in the recalcitrance of burn wound infections, in wound dressing failure and burn wound sepsis (Kennedy et al., 2010; Brandenburg et al., 2019; Moyano et al., 2019). This model can also be used to study biofilm formation at the burn wound site and determine the pathogenicity of high biofilm forming strains (Figure 4B). This invertebrate model could also be used to address the complex question of what represents a burn wound infection and what represents the natural microbial flora of a burn wound. It also has the potential to act as a robust drug discovery platform, by facilitating *in vivo* testing at a throughput level that cannot be achieved in traditional mammalian models. This invertebrate burn wound model, while not a complete replacement for mammalian models of burn trauma and wound infection, does offer a viable alternative that is low cost, less ethically challenging and can facilitate higher power statistical analysis.

## Data Availability Statement

The datasets generated for this study are available on request to the corresponding author.

## Author Contributions

RM, DW, FS, and HA conceived the study. EM, YS, and RM performed the experiments and data analysis. EM and RM wrote the manuscript with inputs from all other authors. EM and YS have contributed equally to the work.

## Conflict of Interest

The authors declare that the research was conducted in the absence of any commercial or financial relationships that could be construed as a potential conflict of interest.
