# Supplementary material for: An Invertebrate Burn Wound Model That Recapitulates the Hallmarks of Burn Trauma and Infection Seen in Mammalian Models
Source: Front Microbiol. 2020 Jun 3;11:998. doi: 10.3389/fmicb.2020.00998 (PMC7283582; doi:10.3389/fmicb.2020.00998)
Supplement: Supplementary file 1 [file Data_Sheet_1.PDF]

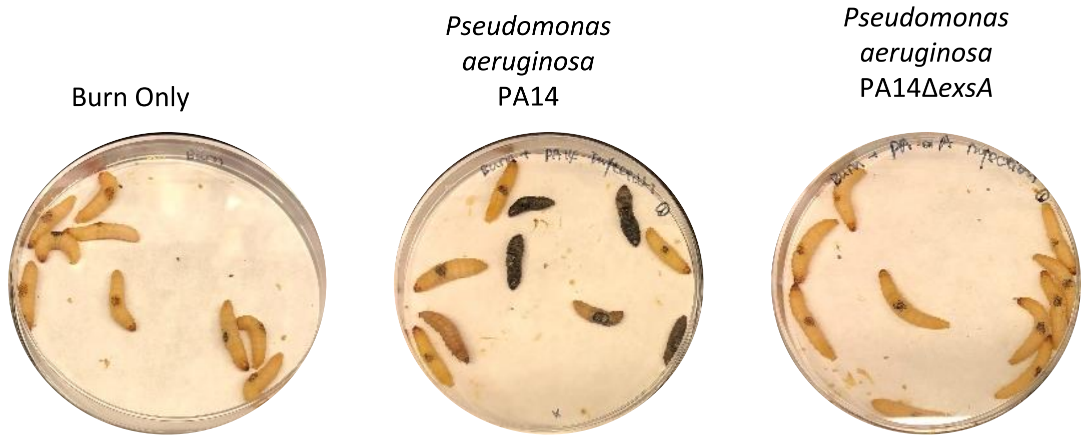

Supplementary Figure 1: *Larvae at 24 hours post burn:* Representative images of larvae 24 hours post burn.
